# Isolation and Genomic Characterization of a Chinese Genotype C Bovine Parainfluenza Virus Type 3 from Cattle and Its Pathogenicity in C57BL/6 Mice

**DOI:** 10.3390/ani14030463

**Published:** 2024-01-31

**Authors:** Jing Chen, Yangyang Qiu, Pan Xiong, Zhijie Wang, Nengzhang Li, Chao Ye, Yuanyi Peng

**Affiliations:** College of Veterinary Medicine, Southwest University, Chongqing 400715, China

**Keywords:** bovine parainfluenza virus type 3, pathogenicity, complete sequence, phylogenetic analysis, genotype C

## Abstract

**Simple Summary:**

Bovine parainfluenza virus type 3 (BPIV-3) is a common respiratory pathogen associated with bovine respiratory disease. BPIV-3 has spread worldwide; however, data on the prevalence and genetic characteristics of BPIV-3 are still limited. In this study, we isolated and characterized the pathogenicity and genetic diversity of a genotype C strain of BPIV-3 (SC) from cattle in China. Infection experiments indicated that the BPIV-3 SC showed a certain degree of pathogenicity in C57BL/6 mice. Genomic sequencing and phylogenetic analysis indicated that our isolated strain was a genotype C strain circulating in China and that SC clustered in the same large clade consisting of a population of Chinese genotype C strains but was found to be different from the other strains upon further differentiation. Meanwhile, 70 nt mutations were found between SC and other Chinese genotype C strains, and 13 of the 70 nt mutations resulted in aa mutations in HN, P, and L genes. The full characterization of the divergent BPIV-3 strain will contribute to an understanding of the prevalence and evolution of BPIV-3 in China and support future molecular diagnoses and efficient vaccine studies.

**Abstract:**

Bovine parainfluenza virus type 3 (BPIV-3), also known as bovine respirovirus 3, is a common respiratory pathogen associated with bovine respiratory disease (BRD). BPIV-3 has currently circulated worldwide; however, data on the prevalence and genetic characteristics of BPIV-3 are still scarce and limited. In this study, the BPIV-3 strain SC was identified and isolated from cattle presenting with clinical signs of BRD in China. Animal experiments indicated that BPIV-3 SC can successfully infect C57BL/6 mice and induce weight loss, lung inflammatory cell infiltration, and inflammatory cytokine expression in mice. In addition, the complete genome of BPIV-3 SC was obtained using next-generation sequencing and was 15,473 bp in length. Phylogenetic analysis indicated that BPIV-3 SC belonged to genotype C, which clustered in the same large clade consisting of a population of Chinese genotype C strains but was found to be different from the other strains upon further differentiation. Compared to other Chinese genotype C strains, the BPIV-3 SC showed 70 unique nucleotide mutations and 13 unique amino acid mutations in the HN, P, and L proteins, suggesting a unique genetic evolution of BPIV-3 SC. In conclusion, we isolated and characterized a differential Chinese genotype C BPIV-3, which contributed to an understanding of the prevalence and evolution of BPIV-3 in China.

## 1. Introduction

Bovine respiratory disease (BRD) is the most common and economically impactful disease of feedlot cattle [1], which can cause acute respiratory disease or chronic and prolonged intractable diseases in cattle [2]. BRD is considered a disease complex that is induced by the interaction of a number of viral and/or bacterial pathogens in the respiratory tracts of cattle [3,4]. For diagnosis of this disease, the observation of clinical signs is the most common method to identify BRD; however, the identification of etiologic agents associated with BRD based on clinical observation is typically not possible. Additionally, necropsy, molecular, and biochemical diagnoses are also available for the diagnosis of BRD, the combination of which can be used for the identification of BRD-associated agents [5]. Bovine parainfluenza virus 3 (BPIV-3) is one of the most important pathogens associated with BRD, and its predisposing role in the onset of BRD has been well studied in different experimental studies [6]. Although clinical signs of BPIV-3 infection are usually mild and characterized as fever, nasal discharge, and dry cough, it can be complicated by coinfection with other respiratory pathogens. Therefore, BPIV-3 is considered an important pathogen in enzootic pneumonia in calves and BRD in feedlot cattle, which seriously threatens the development of the global cattle industry [7]. 

BPIV-3 is also known as bovine respirovirus 3 and belongs to the *Respirovirus* genus in the *Paramyxoviridae* family [8]. The genome of BPIV-3 is a non-segmented, single-stranded, negative-sense RNA of approximately 15.4 kilobases in length, mainly encoding six large open reading frames (ORFs). From 5′ to 3′ on the positive sense strand, the viral ORFs are presented in the following order: the nucleocapsid protein (NP), phosphoprotein (P), matrix protein (M), fusion protein (F), hemagglutinin-neuraminidase protein (HN), and large polymerase protein (L) [9]. To date, three genotypes (A, B, and C) have been described according to the phylogenetic analysis studies. Genotype A has been reported in the United States, Australia, China, Argentina, Japan, and Egypt [9,10,11,12,13,14,15]. Genotype B was originally isolated in Australia [11] and has been currently detected in Australia, the United States, Argentina, and China [9,11,12,16]. Genotype C has been reported in China, South Korea, the United States, and Japan [9,17,18,19]. Hence, all three genotypes have been reported in China. Meanwhile, a seroepidemiological study of BPIV-3 in 12 provinces in China showed that the positivity rate of the BPIV-3 antibody was higher than 50% in all provinces except Jiangxi province (27.8%), indicating that BPIV-3 is currently prevalent in China [20]. 

In this study, we isolated and characterized the pathogenicity and genetic diversity of a genotype C strain of BPIV-3 from cattle in China. Infection experiments indicated that the BPIV-3 SC showed a certain degree of pathogenicity in C57BL/6 mice. Genomic sequencing and phylogenetic analysis indicated that our isolated strain SC was a differential genotype C strain circulating in China, which clustered in the same large clade consisting of a population of Chinese genotype C strains but was found to be different from the other strains upon further differentiation. Meanwhile, genome comparisons between SC and other Chinese genotype C strains revealed 70 nucleotide (nt) point mutations, and 13 mutations were non-synonymous, leading to changes in the amino acid (aa) composition of HN, P, and L genes. The full characterization of the differential BPIV-3 strain will contribute to an understanding of the prevalence and evolution of BPIV-3 in China and support future molecular diagnoses and efficient vaccine studies.

## 2. Materials and Methods

### 2.1. Clinical Samples and Etiological Examinations

In January 2022, a suspected outbreak of BRD occurred in a cattle breeding farm in Sichuan province in China. The diseased cattle showed similar clinical signs, such as depression, cough, dyspnea, fever, nasal discharge, diarrhea, and sporadic bloody stool. With the owners’ consent, a total of 15 nasal swabs from symptomatic cattle were collected and immediately transported to our laboratory at a low temperature for etiological examinations. DNA/RNA were extracted from the clinical samples using the viral genomic DNA/RNA extraction kit (Tiangen Biochemical Technology, Beijing, China) according to the manufacturer’s instructions. The RNA samples were subjected to cDNA synthesis using PrimeScript Master Mix (TAKARA, Dalian, China). Then, the specific PCR was used to screen BRD-associated viruses with primers in Table 1.

### 2.2. Virus Isolation

The supernatant of BPIV-3 positive samples was filtered and inoculated on monolayers of Madin–Darby Bovine Kidney (MDBK) cells. After 1 h adsorption, the inoculum was removed and replaced with growth medium supplemented with 2% FBS. The cells were then incubated at 37 degrees and observed daily for typical cytopathic effects (CPEs) caused by BPIV-3. 

Viruses in the cell supernatant were then prepared for plaque purification. Briefly, MDBK cells at 80–90% confluence were incubated with 10-fold dilutions of virus for 1 h at 37 degrees. The cells were then washed with sterilized PBS and overlaid with MEM containing 1% FBS and 1% agarose and then incubated at 37 degrees in a 5% CO_2_ incubator. After 3–4 days, plaques were picked for the next round of purification. After three rounds of purification, the resulting virus was designated as SC and used for subsequent experiments. To confirm the SC strain isolated in MDBK cells, RNA from virus-infected supernatant was extracted using TRIzol reagent (Invitrogen, Waltham, MA, USA) and then tested using the specific RT-PCR method. Two pairs of primers, BPIV-3-F1 (GAATGACTCATGATAGAGGTAT) and BPIV-3-R1 (AGGACAACCAGTTGTATTACAT) and BPIV-3-F2 (GCTCTTCTCTTTTTGTCCCATTCTT) and BPIV-3-R2 (AACCCCTTCCTCA ATCCTGATATAC), were used here to confirm the presence of BPIV-3 SC with the corresponding PCR amplicons of 647 bp and 422 bp, respectively [21,25].

### 2.3. Immunofluorescence Test

The BPIV-3 strain SC (MOI = 1) was inoculated on monolayers of MDBK cells in 48-well plates and incubated for 36 h at 37 °C in a humidified atmosphere with 5% CO_2_. Then, the supernatant was removed and the cells were washed three times with PBS. Next, the cells were fixed with 4% paraformaldehyde (Sango Biotech, Shanghai, China) for 30 min, permeabilized with 0.1% Triton X-100 for another 5 min at room temperature, and washed three times with PBS. Subsequently, the cells were blocked with 5% bovine serum albumin for 1 h and washed three times with PBS. The cells were then incubated with anti-BPIV-3 polyclonal antibodies conjugated to fluorescein isothiocyanate (VMRD) for 2 h at room temperature and washed three times with PBS. Next, the cells were incubated with DAPI at 37 °C for 5 min and washed three times with PBS. Then, an anti-fluorescence quenching agent (Solarbio, Beijing, China) was added, and observation was performed using an inverted fluorescence microscope (Olympus, Tokyo, Japan).

### 2.4. Electron Microscopy

Briefly, the cell supernatant of BPIV-3-infected MDBK cells was collected and centrifuged at 8000× *g* for 15 min to remove cell debris. The new supernatant was then centrifuged in a 100 kDa ultrafiltration tube (UFC910024, Millipore, Burlington, MA, USA) at 5000× *g* at 4 °C for 30 min and concentrated to 2 mL. Afterward, the viral suspension was negatively stained with 1% phosphotungstic acid and was then added to grids containing a carbon-coated Formvar supporting film for 5 min. Finally, the samples were visualized using an electron microscope (JEM-1400 FLASH, JEOL, Tokyo, Japan) at 80 kV.

### 2.5. Illumina Sequencing

The complete genome sequencing of BPIV-3 SC was conducted at Shanghai Tanpu Biotechnology Co., Ltd. (Shanghai, China). Briefly, the viral RNA was extracted from the culture supernatant of BPIV-3 using TRIzol reagent and prepared for next-generation sequencing. Then the RNA sample was fragmented and subjected to random reverse transcription for cDNA synthesis. Sequencing linkers were connected to both ends of the obtained cDNA fragments. Subsequently, after performing bridge PCR amplification, the cDNA library was sequenced on an Illumina Novaseq6000 platform using a pair-end 150 bp sequencing strategy. Then, de novo assembly was performed using SPAdes v3.14.1. The extracted assembled scaffolds limited the minimum contig length to 100 bases, with the best BLAST hits in the nucleotide database. Finally, the complete sequence of BPIV-3 SC was obtained and annotated on the basis of those annotations of the BPIV-3 genomic sequences in the GenBank database and then submitted to GenBank.

### 2.6. Genomic Characterization and Phylogenetic Analysis

To characterize the overall genetic variation of the genome of BPIV-3, the genome of BPIV-3 SC was aligned with those of the following representative strains: HB2 (GenBank Accession No. OP718793), XJ20055-3 (GenBank Accession No. OM632676), SX2021 (GenBank Accession No. ON804787), XJA13 (GenBank Accession No. KU198929), SD0835 (GenBank Accession No. HQ530153), and SX6 (GenBank Accession No. OP718797); this was performed using the LAGAN genomics analysis tool deposited in mVista (https://genome.lbl.gov/vista/mvista /submit.shtml accessed on 7 November 2023). To identify the unique differences between BPIV-3 SC and the other Chinese strains from the different phylogenetic clusters of genotype C, the corresponding nucleotide/amino acid sequence alignments with the representative isolates (HB2, XJ20055-3, SX2021, XJA13, SD0835, and SX6) were generated using MEGA 5.2 for sequence comparisons.

Phylogenetic analysis was performed with BPIV-3 SC and 54 BPIV-3 strains deposited in GenBank. Sequences were aligned using the web version of MAFFT (https://mafft.cbrc.jp/alignment/server/index.html accessed on 25 October 2023). The phylogenetic tree was constructed on the basis of the neighbor-joining method in MEGA 5.2 with 1000 bootstrap repetitions.

### 2.7. Animal Experiments

To understand the pathogenicity of this novel BPIV-3 strain, a total of 40 specific pathogen-free C57BL/6 mice at 5–6 weeks of age were randomly separated into an infected group (20 mice) and a control group (20 mice), and all mice were maintained under specific pathogen-free conditions with free access to drinking water and food. Under anesthesia, mice in the infected group were inoculated with 40 μL of the virus (10^7^ TCID_50_/mL) via the intranasal route, and mice in the control group were inoculated with equal volumes of DMEM. Then, every 4 mice in the infected group and every 4 mice in the control group were euthanized at different timepoints (1, 2, 3, 5, and 7 days post-infection). Lung samples were collected from each mouse, and one sample from the infected group and one sample from the control group were photographed and then fixed in 4% paraformaldehyde for histopathological examination. Additional lung samples from both the infected and control groups were homogenized for the detection of major inflammatory cytokines via ELISA and virus detection via RT-qPCR, respectively. For the ELISA assay, concentrations of interleukin-1β (IL-1β), interleukin-6 (IL-6), and tumor necrosis factor alpha (TNF-α) were measured using the corresponding ELISA kits (Invitrogen) according to the manufacturer’s instructions. Plates were read using a microtiter plate reader at 450 nm. For the RT-qPCR assay, RNA was extracted using TRIzol reagent, and reverse transcription was performed with Takara PrimeScript RT Master Mix according to the manufacturer’s instructions. Primers and probes for the subsequent qPCR were as follows: F (5′-AGCTGGTGGAGCTGTTATTC-3′), R (5′-GTGCATGCTGCTTCT CATTATC-3′), and probe (FAM-TTGCCCTTGGTCCCTCAATAACAGATG-BHQ1). The qPCR amplifications were performed in duplicate wells using the following program: 95 °C for 30 s, followed by 40 cycles of 95 °C for 5 s and 60 °C for 30 s. Meanwhile, the PCR product that was amplified from the cDNA template of BPIV-3 with the above primers was cloned into the pMD-19T vector (TAKARA, Dalian, China) to obtain the standard plasmid. Then the standard plasmids of different concentrations were used to generate standard curves and quantify copies of the cDNA samples. The procedures for the animal experiments were approved by the Institutional Animal Care and Use Committee of Southwest University, Chongqing, China (LAC2023-1-0412).

## 3. Results

### 3.1. BPIV-3 Detection, Isolation, and Identification

**Figure 1:**
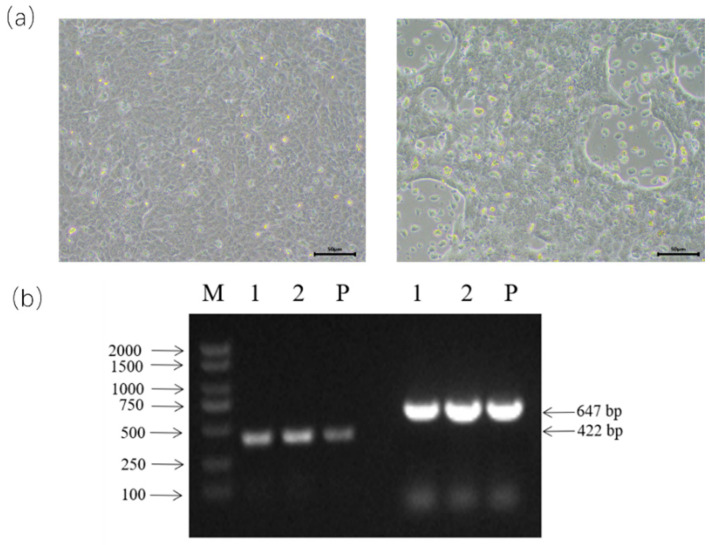
(**a**) The images of mock (**left**) and BPIV-3-infected (**right**) MDBK cells. (**b**) PCR confirmation of BPIV-3 in the cell supernatant. Lane M was the DNA marker; lanes 1 and 2 were virus-infected cell supernatants for BPIV-3 detection; lane P was the positive control for BPIV-3 detection.

In total, 15 nasal swab samples from diseased cattle were pooled into five samples and tested to be BPIV-3 positive using an RT-PCR assay (Figure S1), while other BRD-associated viruses, such as BVDV, BCoV, BRSV, BRV, BAdV-3, and BHV-1, were not detected in these samples. Then, the positive samples were inoculated into MDBK cells, and one of the inoculated cells showed obvious CPE at 48 h post-infection after three generations of blind passaging (Figure 1a); by contrast, no CPE was observed in the control cells (Figure 1a). The viruses were purified via three consecutive rounds of plaque purification in MDBK cells and then confirmed using the specific RT-PCR assay (Figure 1b). 

**Figure 2:**
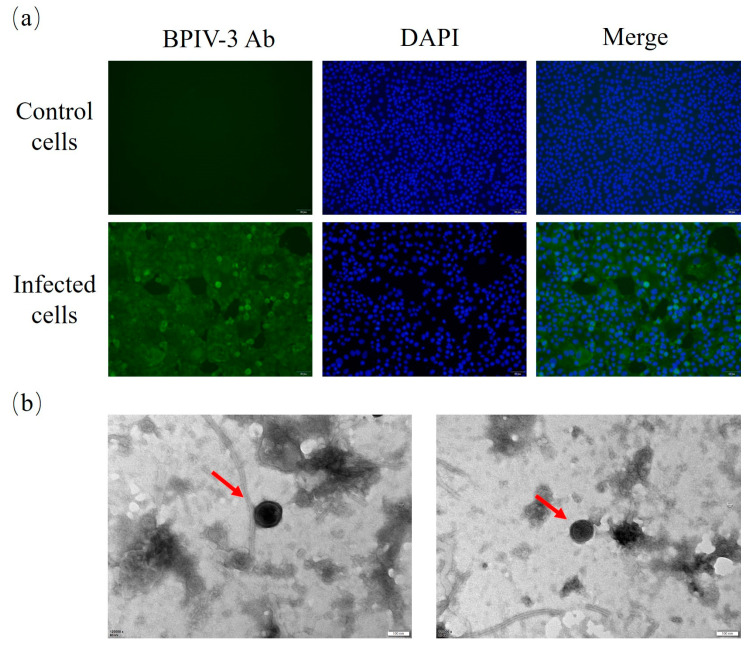
(**a**) Immunofluorescence assay of MDBK cells infected with BPIV-3 SC (MOI = 1) at 36 h post-infection. The specific fluorescence was observed in BPIV-3-infected MDBK cells, and mock-infected MDBK cells were used as the negative control. (**b**) Paramyxovirus-like particles were observed under transmission electron microscopy (bar = 100 nm). The red arrows point to the virus particles.

Furthermore, an immunofluorescence assay showed that the specific fluorescence signal could be detected in the virus-infected cytoplasm, while no virus-specific fluorescence signal was found in the control cells (Figure 2a). Meanwhile, electron microscopy showed that the virus particle was about 100 nm in diameter, with envelope and filamentous structures outside the virus (Figure 2b), which was similar to those of the paramyxovirus members.

### 3.2. Pathogenicity of BPIV-3 SC in C57BL/6 Mice

**Figure 3:**
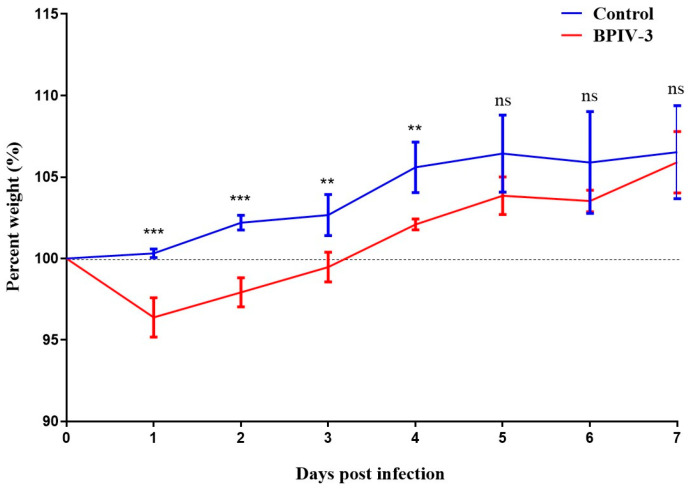
Body weight changes of mice at different times after BPIV-3 infection. Data are presented as averages ± SD and analyzed using Student’s *t* tests for two-group comparisons. Statistical significance is shown as **, *p* < 0.01; ***, *p* < 0.001; and ns, no significance.

Mice in the infected group showed mild clinical symptoms, such as lethargy, weight loss, and loss of appetite at 1–3 days post-infection, and no typical respiratory symptoms were observed throughout the observation period. Compared with the control group, mice in the infected group showed obvious body weight loss from 1 to 3 days post-infection and gradually recovered and caught up with the control group on day 7 (Figure 3).

**Figure 4:**
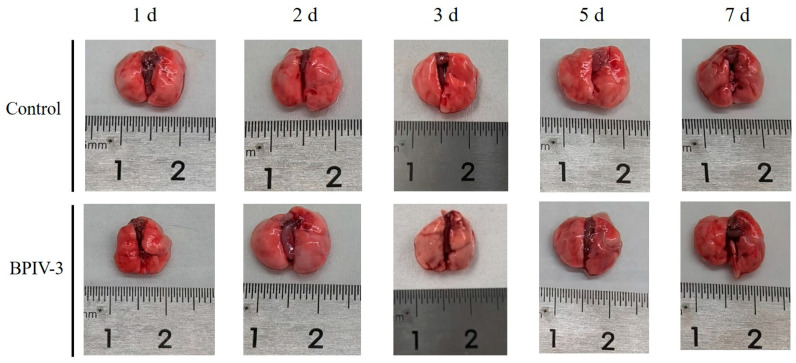
Gross lesions in lungs of C57BL/6 mice after infection with BPIV-3 SC.

**Figure 5:**
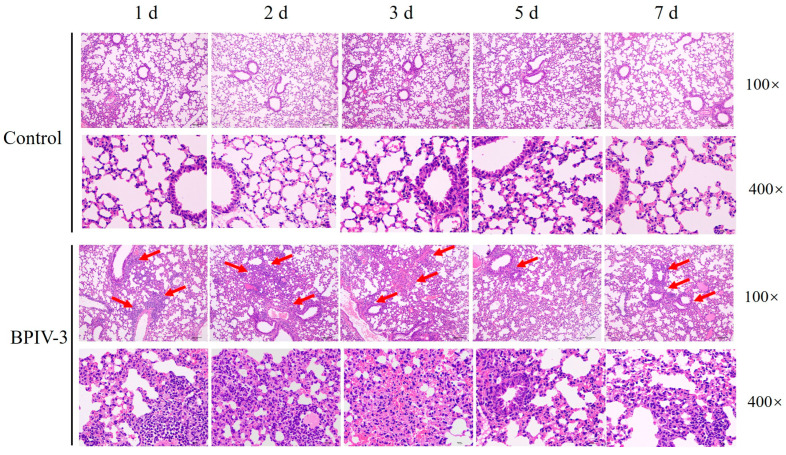
Lung tissues of mice after hematoxylin and eosin (H&E) staining. At 1, 2, 3, 5, and 7 days post-infection, alveolar septal thickening, serous exudation, and lymphocyte infiltration were observed in lung sections of BPIV-3-infected mice. The red arrows point to the areas of lymphocyte infiltration.

Compared with the control mice, gross lesions showed that the lungs from the infected mice increased in size, and mild pulmonary edema at 2 days post-infection was observed (Figure 4). Histopathological examination showed that typical histopathological changes, such as alveolar septal thickening, serous exudation, and lymphocyte infiltration, were observed in infected mice at 1–3 days post-infection, and then these lung injuries were significantly alleviated at 5 days post-infection (Figure 5). 

**Figure 6:**
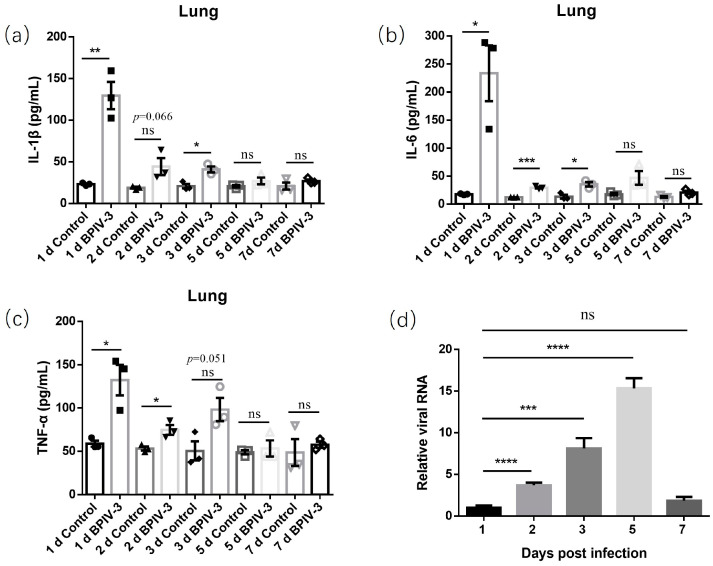
Levels of proinflammatory cytokines IL-1β (**a**), IL-6 (**b**), and TNF-α (**c**) in lung tissue homogenates were evaluated using ELISA. Replication kinetics of BPIV-3 SC in lungs of the infected mice was evaluated using RT-qPCR (**d**), and copy number ratios of samples from groups at 2, 3, 5, and 7 dpi and samples from groups at 1 dpi are shown. Data are presented as averages ± SEM and analyzed using Student’s *t* tests for two-group comparisons. Statistical significance is shown as *, *p* < 0.05; **, *p* < 0.01; ***, *p* < 0.001; ****, *p* < 0.0001; and ns, no significance.

Furthermore, levels of proinflammatory cytokines in lung tissue homogenates were evaluated using ELISA. In accordance with the relevant pathological changes, the selected proinflammatory cytokines, including IL-1β, IL-6, and TNF-α, in the infected mice were obviously elevated at 1–3 days post-infection and then maintained a slight upregulation at lower levels at 5 and 7 days post-infection (Figure 6a–c). Meanwhile, the replication kinetics of the virus in lungs of the infected mice was evaluated using RT-qPCR. Similar to previous reports, the BPIV-3 virus could be detected in the lungs of mice. Moreover, the virus could replicate in the lungs of mice, reaching the highest viral load on day 5 and then decreasing on day 7 (Figure 6d). 

### 3.3. Whole Genome Sequencing and Phylogenetic Analysis of BPIV-3

Illumina sequencing generated around 9.8 million reads. After quality control, around 9.1 million clean reads were obtained. After subtracting ribosomal RNAs and host reads, the contig containing the complete genome of BPIV-3 SC was obtained and was 15,473 bp in length. On the basis of those annotations of the BPIV-3 genomic sequences stored in the public database, the complete sequence of the BPIV-3 strain SC was annotated and then submitted to GenBank with accession No. OR520601.

**Figure 7:**
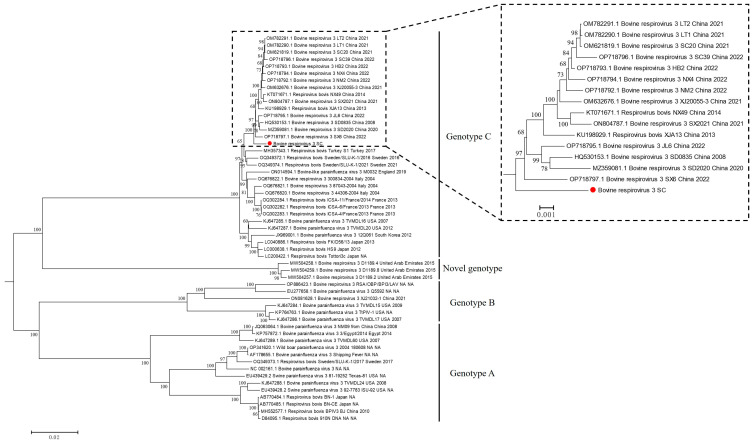
Phylogenetic analysis of BPIV-3 strains based on the complete genome sequences. The neighbor-joining method was used to construct phylogenetic tree, and bootstrap values of 1000 replicates were calculated. BPIV-3 SC was labeled with a red dot.

To investigate the phylogeny of BPIV-3 worldwide, the complete sequence of BPIV-3 SC was aligned to that of 51 parainfluenza virus 3 strains of bovine origin and 3 parainfluenza virus 3 strains of swine origin deposited in the GenBank database. Subsequently, a phylogenetic tree based on the complete sequences of BPIV-3 was constructed using the neighbor-joining method, which showed that the global BPIV-3 strains were separated into four major clades, i.e., genotype A, B, C, and a novel genotype composed of strains from the United Arab Emirates (Figure 7). Moreover, the BPIV-3 strain SC isolated in this study belonged to genotype C and clustered with most of the recent Chinese strains in one large clade; however, it was different from the other Chinese genotype C strains and also formed a small branch by itself (Figure 7). In addition, very few (*n*= 3) Chinese strains were scattered in clades of genotype A and B (Figure 7).

### 3.4. Genomic Characteristics of BPIV-3 SC

**Figure 8:**
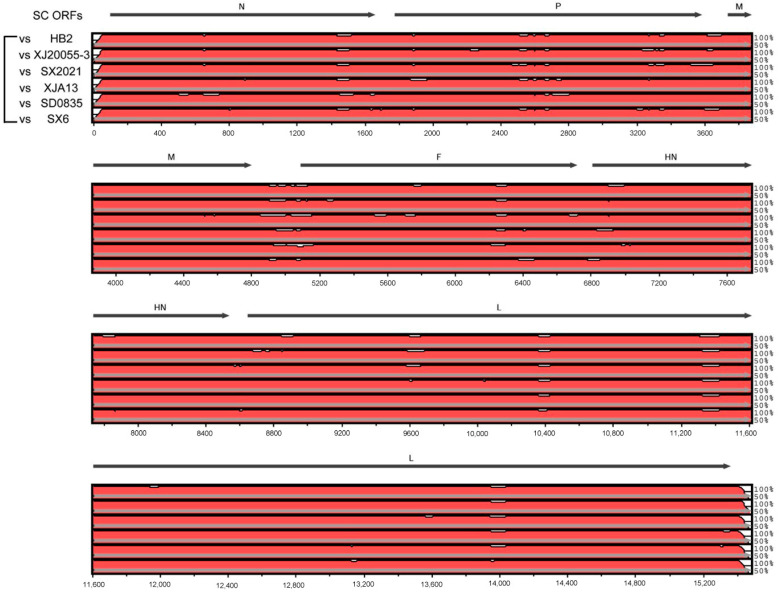
Genomic organization of BPIV-3 and comparison of sequence conservation within the BPIV-3 SC, HB2, XJ20055-3, SX2021, XJA13, SD0835, and SX6 strains. The mVISTA similarity plot showed sequence conservation between SC and the other BPIV-3 strains. Sequence conservation was determined from a multiple sequence alignment, and the conservation score was plotted in a sliding 100 bp window.

To compare the genomic variation of the BPIV-3 strain SC with other circulating Chinese strains, the complete sequence of BPIV-3 SC was aligned with the representative Chinese strains in the same major clade. It showed that the genomes of SC and the other Chinese strains had the same overall genomic composition; however, many hypervariable regions were still observed in the multi-genome alignment of SC and the other Chinese strains. In general, differences between SC and the other closely related Chinese strains were distributed throughout the entire genomes but were predominantly found in regions of P, F, and L ORFs (Figure 8). Meanwhile, the genome of strain SC shared 98.9–99.1% nt identity with the other Chinese strains in the same major clade and shared 97.3–99.1% nt identity with all the complete genomes of genotype C BPIV-3 strains in GenBank (Figure S2). Furthermore, numerous nt mutations were observed between the BPIV-3 SC strain and the other Chinese genotype C strains, including 7 sites in the N gene, 14 sites in the P gene, 3 sites in the M gene, 8 sites in the F gene, 5 sites in the HN gene, and 33 sites in the L gene (Table S1). Meanwhile, genome comparisons between SC and the other Chinese genotype C strains revealed that 13 mutations were non-synonymous, leading to changes in the amino acid composition of several genes. Specifically, four aa substitutions in the P gene, three aa substitutions in the HN gene, and six aa substitutions in the L gene were observed (Table S1).

## 4. Discussion

BPIV-3 can cause variable symptoms, from asymptomatic infections to severe respiratory illness, in cattle. Generally, as one of the major causative pathogens of BRD, mixed infections of BPIV-3 with other BRD-associated pathogens can cause more severe clinical symptoms and economic losses in cattle. In China, BPIV-3 has been shown to be prevalent in at least 12 provinces in China, as demonstrated by a seroepidemiological study [20]. Moreover, in addition to cattle, infection due to and prevalence of BPIV-3 have also been recently reported in yaks [25], suggesting that the virus is an important threat to the cattle and yak breeding industries in China.

In this study, we detected BPIV-3 infections in diseased cattle in Southwest China using RT-PCR, then isolated a BPIV-3 strain using Madin–Darby bovine kidney cells, and subsequently confirmed the identity of this virus using RT-PCR, an immunofluorescence test, and electron microscopy. Furthermore, consistent with previous studies [26,27], animal experiments showed that the virus was pathogenic to C57BL/6 mice and can induce weight loss, lung inflammatory cell infiltration, and inflammatory cytokine expression in mice, indicating that mice can be used as an animal model for the infection process and studies of the pathogenicity of BPIV-3. Hence, it can be expected that using the mouse model instead of cattle will greatly reduce the cost of animal experiments and test cycles and improve the accuracy and repeatability of experiments in future BPIV-3-related studies.

The complete sequence of BPIV-3 SC was obtained using Illumina sequencing and was 15,473 bp in length, which enriched the complete sequence information of BPIV-3 in the GenBank database. Then, phylogenetic analysis based on the complete genomes of BPIV-3 confirmed the presence of three existing genotypes (A, B, and C), and a novel genotype consisted of strains from the United Arab Emirates. Furthermore, as previously reported, bovine-like parainfluenza virus 3 was also found in camels [28], fallow deer [29], swine [30,31], and even wild boar [32], suggesting the complex genetic diversity and potential cross-species infection characteristics of BPIV-3. Consistent with the previous study [16], all the existing Chinese strains in the GenBank database were distributed in the branches of genotype A, B and C, but most of the strains and the BPIV-3 SC isolated in this study were distributed in genotype C. Interestingly, BPIV-3 SC and other Chinese strains in genotype C were closely related and clustered together, suggesting a close evolutionary relationship between Chinese genotype C strains. Moreover, although the difference seems small, BPIV-3 SC formed a small evolutionary branch that separated it from other Chinese genotype C strains, suggesting that SC might be a differential Chinese genotype C strain.

The similarity analysis showed that the genome of strain SC exhibited 0.9–1.1% divergence with the other Chinese strains in the same major clade at the nucleotide level. Many variable sites (nt mutations) were found in the multi-genome alignment of SC and other Chinese genotype C strains, which were predominantly located in regions of the P, F, and L genes. Specifically, a total of 7 sites in the N gene, 14 sites in the P gene, 3 sites in the M gene, 8 sites in the F gene, 5 sites in the HN gene, and 33 sites in the L gene were observed. Furthermore, genome comparisons of SC and other Chinese genotype C strains revealed that 13 mutations were non-synonymous, leading to changes in the amino acid composition of several genes, including four aa substitutions in the P gene, three aa substitutions in the HN gene, and six aa substitutions in the L gene. These altered aa sites may affect the conformation and antigenicity of the relevant proteins; however, this needs to be further explored in future studies. 

## 5. Conclusions

In conclusion, we isolated a Chinese genotype C BPIV-3 from cattle in China and characterized its pathogenicity and genetic diversity. The pathogenicity results indicated that the BPIV-3 SC in this study possessed a certain degree of pathogenicity in C57BL/6 mice, suggesting that SPF animals like mice may be a feasible model for the study of BPIV-3. Phylogenetic analysis indicated that our isolated strain is a divergent genotype C strain circulating in China and that SC was clustered within the population of Chinese genotype C strains but was found to be different from the other strains upon further differentiation. Meanwhile, 70 nt mutations were observed between SC and other Chinese genotype C strains, 13 of which resulted in aa mutations in the HN, P, and L genes. The characterization of BPIV-3 SC will contribute to an understanding of the prevalence and variation of BPIV-3 in China, which may support future molecular diagnoses, molecular epidemiological investigations, and efficient vaccine studies of BPIV-3.

## Figures and Tables

**Table 1:** Primers used for bovine respiratory disease (BRD)-associated virus detection.

Pathogens	Sequence (5′-3′)	Length of Amplicon (bp)	References
BVDV	GGTAGCAACAGTGGTGAGTTC	130	[21]
	CTCAGGTTAAGATGTGCTGTG		
BCoV	ACGTTCTTTTAAAACAGCCGATG	409	[22]
	TGCCAGAACAAGACTAGCAA		
BRSV	TATGCTATGTCCCGATTGG	600	[21]
	ACTGATTTGGCTAGTACACCC		
BRV	GGTAGCGGCGTTATTTCC	407	[23]
	CGCCATCTGAGTGATTACTC		
BPIV-3	GCATCACAAACTCCGCAATAT	1048	[17]
	TGCTTGATTTTTCCGACTCCT		
BADV-3	CTCCTGGGTCCTGGCCTTAGTT	1182	[24]
	AGTGTTTGTGGGTAAAGGGCAATAG		
BHV-1	GCTCGCCAACTTCTTTCAGGG	306	[21]
	GCGTCAAACTCCTCCTCTTCCTC		

## Data Availability

The sequencing results in this study have been submitted to the GenBank database under accession No. OR520601.

## Supplementary Materials

The following supporting information can be downloaded at https://www.mdpi.com/article/10.3390/ani14030463/s1, Figure S1: Detection of BPIV-3 from 15 nasal swab samples by RT-PCR; Figure S2: The nucleotide similarity comparison based on the complete genomes between BPIV-3 SC strain and other related strains; Table S1: Unique SNPs and amino acid substitutions in each gene of SC versus other representative Chinese Genotype C strains.
