# Supplementary material for: Isolation and Genomic Characterization of a Chinese Genotype C Bovine Parainfluenza Virus Type 3 from Cattle and Its Pathogenicity in C57BL/6 Mice
Source: Animals (Basel). 2024 Jan 31;14(3):463. doi: 10.3390/ani14030463 (PMC10854764; doi:10.3390/ani14030463)
Supplement: Supplementary file 1 [file animals-14-00463-s001.zip › animals-2809165-supplementary.pdf]

Figure S1. Detection of BPIV-3 from 15 nasal swab samples by RT-PCR. Lane M was the DNA marker; lanes 1-5 were 5 pooled samples (3 samples in one pool); lane N was negative control for BPIV-3 detection.

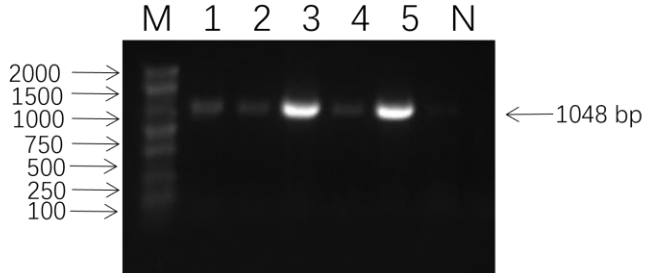

Figure S2. The nucleotide similarity comparison based on the complete genomes between BPIV-3 SC strain and other related strains. (a) Percent identity among SC and the other Chinese strains in the same major clade. (b) Percent identity among SC and all the other genotype C BPIV-3 strains in GenBank.

(a)

Percent Identity

	1	2	3	4	5	6	7	8	9	10	11	12	13	14	15	16		
1		98.9	98.9	98.9	98.9	99.0	98.9	98.9	99.0	98.9	98.9	99.0	99.0	99.1	99.0	99.1	1	SC
2	1.0		100.0	100.0	99.8	99.9	99.8	99.8	99.6	99.6	99.5	99.5	99.4	99.2	99.1	99.3	2	LT2
3	1.0	0.0		100.0	99.8	99.9	99.8	99.8	99.6	99.6	99.5	99.5	99.4	99.2	99.1	99.3	3	LT1
4	1.0	0.0	0.0		99.8	99.9	99.8	99.8	99.6	99.6	99.5	99.5	99.4	99.2	99.1	99.3	4	SC20
5	1.1	0.2	0.2	0.2		99.8	99.7	99.7	99.6	99.5	99.5	99.4	99.3	99.2	99.0	99.2	5	SC39
6	1.0	0.1	0.1	0.1	0.2		99.8	99.8	99.6	99.7	99.6	99.5	99.4	99.3	99.1	99.3	6	HB2
7	1.1	0.2	0.2	0.2	0.3	0.2		99.8	99.5	99.6	99.6	99.5	99.3	99.2	99.1	99.2	7	NX4
8	1.0	0.2	0.2	0.2	0.3	0.2	0.2		99.6	99.7	99.7	99.5	99.4	99.2	99.1	99.3	8	NM2
9	1.0	0.3	0.3	0.3	0.4	0.3	0.4	0.4		99.5	99.5	99.4	99.3	99.2	99.1	99.2	9	XJ20055- 3
10	1.0	0.4	0.4	0.4	0.5	0.3	0.4	0.3	0.4		99.7	99.5	99.3	99.3	99.1	99.2	10	NX49
11	1.1	0.5	0.5	0.5	0.5	0.4	0.4	0.3	0.4	0.3		99.4	99.2	99.2	99.0	99.2	11	SX2021
12	0.9	0.5	0.5	0.5	0.6	0.5	0.5	0.5	0.5	0.5	0.6		99.4	99.3	99.2	99.3	12	XJA13
13	1.0	0.7	0.7	0.6	0.7	0.6	0.7	0.6	0.7	0.7	0.8	0.6		99.5	99.5	99.3	13	JL6
14	0.9	0.8	0.8	0.8	0.8	0.7	0.8	0.8	0.7	0.7	0.8	0.7	0.5		99.5	99.3	14	SD0835
15	1.0	0.9	0.9	0.9	1.0	0.9	0.9	0.9	0.9	0.9	1.0	0.8	0.5	0.5		99.3	15	SD2020
16	0.9	0.7	0.7	0.7	0.8	0.7	0.8	0.7	0.7	0.8	0.8	0.7	0.7	0.6	0.7		16	SX6
	1	2	3	4	5	6	7	8	9	10	11	12	13	14	15	16		

(b)

Percent Identity

	1	2	3	4	5	6	7	8	9	10	11	12	13	14	15	16	17	18	19	20	21	22	23	24	25	26	27	28	29	30	31	32		
1		99.1	97.3	97.9	97.7	98.9	99.0	97.9	97.8	97.8	98.2	99.0	98.9	99.0	98.9	98.9	97.8	98.9	98.9	99.0	98.9	99.0	98.9	99.1	98.1	98.0	98.0	98.3	98.2	97.9	98.0	98.3	1	SC
2	0.9		97.7	98.2	98.0	99.3	99.3	98.1	98.0	98.0	98.5	99.5	99.2	99.2	99.2	99.2	98.0	99.2	99.2	99.3	99.2	99.5	99.2	99.4	98.4	98.2	98.2	98.6	98.5	98.0	98.3	98.4	2	SD0835
3	2.7	2.4		98.4	98.2	97.5	97.5	98.5	98.5	98.5	97.5	97.5	97.5	97.4	97.5	97.5	97.3	97.5	97.5	97.5	97.5	97.5	97.4	97.6	97.6	97.4	97.4	97.6	97.6	97.3	97.5	97.6	3	12Q061
4	2.1	1.9	1.6		98.7	98.0	98.1	98.8	98.7	98.7	98.1	98.0	98.0	98.0	98.0	98.0	97.9	98.0	98.0	98.0	98.0	98.1	97.9	98.1	98.1	97.9	97.9	98.2	98.1	97.8	98.0	98.2	4	TVMDL16
5	2.4	2.1	1.9	1.3		97.8	97.9	98.6	98.5	98.5	97.9	97.8	97.8	97.7	97.8	97.8	97.7	97.8	97.8	97.8	97.8	97.9	97.7	97.9	97.9	97.7	97.7	98.0	97.9	97.6	97.8	98.0	5	TVMDL20
6	1.0	0.7	2.6	2.0	2.2		99.5	98.0	97.9	97.9	98.3	99.1	99.6	99.5	99.6	99.6	97.9	99.8	99.7	99.7	99.6	99.3	99.5	99.3	98.2	98.0	98.0	98.5	98.4	97.9	98.1	98.2	6	NX49
7	0.9	0.7	2.5	2.0	2.2	0.5		98.0	98.0	98.0	98.4	99.2	99.5	99.4	99.5	99.5	97.9	99.4	99.5	99.6	99.5	99.4	99.4	99.3	98.3	98.1	98.1	98.5	98.4	97.9	98.1	98.3	7	XJA13
8	2.1	1.9	1.5	1.2	1.5	2.1	2.0		99.5	99.5	98.1	98.0	98.0	97.9	97.9	98.0	97.8	97.9	97.9	98.0	97.9	98.0	97.9	98.1	98.1	97.9	97.9	98.2	98.1	97.8	98.0	98.2	8	HS9
9	2.2	2.0	1.5	1.3	1.5	2.1	2.0	0.4		99.4	98.1	97.9	97.9	97.8	97.9	97.9	97.8	97.9	97.9	97.9	97.9	98.0	97.8	98.0	98.1	97.9	97.9	98.1	98.0	97.8	98.0	98.1	9	FK/ D56/ 13
10	2.2	2.0	1.5	1.3	1.5	2.1	2.1	0.4	0.5		98.0	97.9	97.9	97.9	97.9	97.9	97.8	97.9	97.9	97.9	97.9	98.0	97.8	98.0	98.1	97.9	97.9	98.2	98.0	97.8	98.0	98.2	10	Tottori3c
11	1.7	1.5	2.5	1.9	2.2	1.7	1.6	2.0	2.0	2.0		98.3	98.3	98.3	98.3	98.3	98.1	98.2	98.3	98.3	98.3	98.4	98.2	98.4	98.3	98.2	98.2	98.6	98.6	98.1	98.3	98.5	11	Turkey_S1
12	1.0	0.5	2.6	2.1	2.3	0.9	0.8	2.1	2.1	2.1	1.7		99.1	99.1	99.1	99.1	97.9	99.1	99.1	99.2	99.1	99.6	99.0	99.3	98.3	98.1	98.0	98.5	98.4	97.9	98.1	98.2	12	SD2020
13	1.0	0.8	2.6	2.1	2.3	0.4	0.5	2.1	2.1	2.1	1.7	0.9		99.6	100.0	100.0	97.8	99.5	99.8	99.9	99.8	99.4	99.8	99.3	98.2	98.0	98.0	98.4	98.3	97.8	98.1	98.2	13	SC20
14	1.0	0.7	2.6	2.0	2.3	0.4	0.5	2.1	2.1	2.1	1.7	0.9	0.3		99.6	99.6	97.8	99.5	99.6	99.6	99.5	99.3	99.6	99.2	98.2	98.1	98.1	98.4	98.3	98.0	98.1	98.3	14	XJ20055- 3
15	1.0	0.8	2.6	2.1	2.3	0.4	0.5	2.1	2.1	2.1	1.7	0.9	0.0	0.3		100.0	97.8	99.5	99.8	99.9	99.8	99.4	99.8	99.3	98.2	98.0	97.9	98.4	98.3	97.8	98.1	98.2	15	LT1
16	1.0	0.8	2.6	2.1	2.3	0.4	0.5	2.1	2.1	2.1	1.7	0.9	0.0	0.3	0.0		97.8	99.5	99.8	99.9	99.8	99.4	99.8	99.3	98.2	98.0	98.0	98.4	98.3	97.8	98.1	98.2	16	LT2
17	2.3	2.0	2.8	2.2	2.3	2.2	2.1	2.2	2.2	2.2	1.9	2.2	2.2	2.2	2.2	2.2		97.8	97.8	97.9	97.8	97.9	97.8	97.9	98.4	98.2	98.2	98.2	98.0	98.1	98.3	98.5	17	M0032
18	1.1	0.8	2.6	2.1	2.3	0.3	0.6	2.1	2.2	2.2	1.8	1.0	0.5	0.4	0.5	0.5	2.3		99.7	99.6	99.6	99.2	99.5	99.2	98.1	97.9	97.9	98.4	98.3	97.8	98.0	98.2	18	SX2021
19	1.0	0.8	2.6	2.1	2.3	0.3	0.5	2.1	2.1	2.1	1.7	0.9	0.2	0.4	0.2	0.2	2.2	0.3		99.8	99.8	99.4	99.7	99.3	98.2	98.0	98.0	98.4	98.3	97.8	98.0	98.2	19	NM2
20	1.0	0.7	2.5	2.0	2.2	0.3	0.5	2.1	2.1	2.1	1.7	0.9	0.1	0.3	0.1	0.1	2.2	0.4	0.2		99.8	99.4	99.8	99.3	98.2	98.0	98.0	98.5	98.4	97.9	98.1	98.3	20	HB2
21	1.1	0.8	2.6	2.1	2.3	0.4	0.5	2.1	2.1	2.2	1.8	0.9	0.2	0.4	0.2	0.2	2.3	0.4	0.2	0.2		99.3	99.7	99.2	98.1	97.9	97.9	98.4	98.3	97.8	98.0	98.2	21	NX4
22	1.0	0.5	2.5	2.0	2.2	0.7	0.6	2.0	2.0	2.1	1.6	0.5	0.6	0.7	0.7	0.7	2.1	0.8	0.6	0.6	0.7		99.3	99.3	98.3	98.1	98.1	98.5	98.4	98.0	98.2	98.3	22	JL6
23	1.1	0.8	2.7	2.1	2.4	0.5	0.6	2.2	2.2	2.2	1.8	1.0	0.2	0.4	0.2	0.2	2.3	0.5	0.3	0.2	0.3	0.7		99.2	98.1	97.9	97.9	98.3	98.3	97.8	98.0	98.1	23	SC39
24	0.9	0.6	2.5	1.9	2.2	0.8	0.7	2.0	2.0	2.1	1.6	0.7	0.7	0.7	0.7	0.7	2.1	0.8	0.7	0.7	0.8	0.7	0.8		98.3	98.1	98.1	98.5	98.4	98.0	98.2	98.3	24	SX6
25	1.9	1.6	2.5	1.9	2.1	1.8	1.8	1.9	1.9	1.9	1.7	1.8	1.9	1.8	1.9	1.9	1.7	1.9	1.9	1.8	1.9	1.7	1.9	1.7		99.8	99.8	98.5	98.4	99.1	99.3	98.8	25	ICSA- 6/ France/ 2013
26	1.9	1.6	2.4	1.9	2.1	1.8	1.8	1.9	1.9	1.9	1.6	1.8	1.9	1.8	1.9	1.9	1.6	1.9	1.9	1.8	1.9	1.7	1.9	1.7	0.0		100.0	98.4	98.2	99.3	99.3	99.0	26	ICSA- 4/ France/ 2013
27	1.9	1.6	2.4	1.9	2.1	1.8	1.8	1.9	1.9	1.9	1.6	1.8	1.9	1.8	1.9	1.9	1.6	1.9	1.9	1.8	1.9	1.7	1.9	1.7	0.0	0.0		98.4	98.2	99.3	99.3	99.0	27	ICSA- 11/ France/ 2014
28	1.6	1.4	2.4	1.8	2.0	1.5	1.5	1.8	1.8	1.8	1.3	1.5	1.5	1.5	1.6	1.5	1.7	1.6	1.6	1.5	1.6	1.5	1.6	1.4	1.5	1.5	1.5		98.8	98.2	98.4	98.6	28	Sweden/ SLU- K- 1/ 2016
29	1.5	1.3	2.3	1.7	1.9	1.4	1.4	1.7	1.7	1.8	1.1	1.4	1.5	1.4	1.5	1.5	1.8	1.5	1.5	1.4	1.5	1.4	1.5	1.4	1.4	1.4	1.4	0.9		98.0	98.3	98.4	29	Sweden/ SLU- K- 1/ 2021
30	2.0	1.7	2.5	2.0	2.2	1.9	1.9	1.9	2.0	2.0	1.6	1.9	2.0	1.9	2.0	2.0	1.7	2.0	2.0	1.9	2.0	1.8	2.0	1.8	0.7	0.7	0.7	1.6	1.5		99.2	98.9	30	44306- 2004
31	1.9	1.7	2.4	1.9	2.1	1.9	1.8	1.9	1.9	1.9	1.6	1.8	1.9	1.8	1.9	1.9	1.6	1.9	1.9	1.8	1.9	1.8	2.0	1.8	0.6	0.6	0.6	1.5	1.4	0.7		99.0	31	87043- 2004
32	1.6	1.4	2.2	1.6	1.8	1.6	1.5	1.6	1.7	1.7	1.3	1.6	1.6	1.6	1.6	1.6	1.4	1.7	1.7	1.6	1.7	1.5	1.7	1.5	1.0	1.0	1.0	1.3	1.2	1.1	0.9		32	300834- 2004
	1	2	3	4	5	6	7	8	9	10	11	12	13	14	15	16	17	18	19	20	21	22	23	24	25	26	27	28	29	30	31	32		

Table S1. Unique SNPs and amino acid substitutions in each gene of SC versus other representative Genotype C Chinese strains

Gene	Loci in the indicated genes of SC	SC	HB2	XJ20055-3	SX2021	XJA13	SD0835	SX6
N	141	G	A	A	A	A	A	A
	408	C	T	T	T	T	T	T
	588	A	G	G	G	G	G	G
	744	A	G	G	G	G	G	G
	1236	A	G	G	G	G	G	G
	1368	T	C	C	C	C	C	C
	1377	A	G	G	G	G	G	G
P	69	T	C	C	C	C	C	C
	150	T	C	C	C	C	C	C
	295	A (I)	G (V)	G (V)	G (V)	G (V)	G (V)	G (V)
	364	G (V)	A (M)	A (M)	A (M)	A (M)	A (M)	A (M)
	491	C (P)	T (L)	T (L)	T (L)	T (L)	T (L)	T (L)
	732	A	G	G	G	G	G	G
	780	T	C	C	C	C	C	C
	860	C (T)	T (I)	T (I)	T (I)	T (I)	T (I)	T (I)
	870	A	G	G	G	G	G	G
	930	A	G	G	G	G	G	G
	975	A	G	G	G	G	G	G
	1452	C	T	T	T	T	T	T
	1540	C	T	T	T	T	T	T
	1749	C	T	T	T	T	T	T

M	433	C	T	T	T	T	T	T
	513	T	C	C	C	C	C	C
	828	C	T	T	T	T	T	T
F	24	G	A	A	A	A	A	A
	177	T	C	C	C	C	C	C
	318	A	G	G	G	G	G	G
	459	C	T	T	T	T	T	T
	663	A	G	G	G	G	G	G
	1158	C	T	T	T	T	T	T
	1197	C	T	T	T	T	T	T
	1587	C	T	T	T	T	T	T
HN	139	A (M)	G (V)	G (V)	G (V)	G (V)	G (V)	G (V)
	390	G (K)	T (N)	T (N)	T (N)	T (N)	T (N)	T (N)
	563	C (T)	A (N)	A (N)	A (N)	A (N)	A (N)	A (N)
	1005	T	C	C	C	C	C	C
	1011	C	T	T	T	T	T	T
L	75	G	T	T	T	T	T	T
	90	G	A	A	A	A	A	A
	242	G (S)	C (T)	C (T)	C (T)	C (T)	T (I)	T (I)
	301	C	T	T	T	T	T	T
	858	C	T	T	T	T	T	T
	967	G (V)	A (I)	A (I)	A (I)	A (I)	A (I)	A (I)
	998	T (I)	C (T)	C (T)	C (T)	C (T)	C (T)	C (T)

1333	A (I)	G (V)	G (V)	G (V)	G (V)	G (V)	G (V)
1428	G	A	A	A	A	A	A
1758	T	C	C	C	C	C	C
2405	G (R)	A (K)	A (K)	A (K)	A (K)	A (K)	A (K)
2640	A	G	G	G	G	G	G
2721	C	T	T	T	T	T	T
2724	C	T	T	T	T	T	T
2730	C	T	T	T	T	T	T
2745	C	T	T	T	T	T	T
2859	C	T	T	T	T	T	T
3030	T	A	A	A	A	A	A
3297	A	G	G	G	G	G	G
3339	G	A	A	A	A	A	A
3450	G	A	A	A	A	A	A
4464	G	A	A	A	A	A	A
4524	G	A	A	A	A	A	A
4896	A	G	G	G	G	G	G
4959	C	T	T	T	T	T	T
5335	A (I)	G (V)	G (V)	G (V)	G (V)	G (V)	G (V)
5349	C	T	T	T	T	T	T
5562	G	A	A	A	A	A	A
5892	A	G	G	G	G	G	G
6037	T	C	C	C	C	C	C
6066	A	G	G	G	G	G	G
6441	A	G	G	G	G	G	G
6696	C	T	T	T	T	T	T
